# Neutrophil percentage-to-albumin ratio is associated with low muscle mass risk in rheumatoid arthritis: evidence from a hospital-based cohort and NHANES validation

**DOI:** 10.3389/fimmu.2026.1881390

**Published:** 2026-07-31

**Authors:** Yuan Zhang, Shanggao Xie, Qice Sun, Kai Chen, Liesu Meng

**Affiliations:** 1 Institute of Molecular and Translational Medicine, and Department of Biochemistry and Molecular Biology, School of Basic Medical Sciences, Xi’an Jiaotong University Health Science Center, Xi’an Jiaotong University, Xi’an, China; 2 Department of Rheumatology, The Second Affiliated Hospital of Zhejiang Chinese Medical University, Hangzhou, China; 3 Center for Plastic and Reconstructive Surgery, Department of Orthopedics, Zhejiang Provincial People’s Hospital (Affiliated People’s Hospital, Hangzhou Medical College), Hangzhou, Zhejiang, China; 4 Department of General Surgery, The Second Affiliated Hospital of Xi’an Jiaotong University, Xi'an, Shaanxi, China; 5 Key Laboratory of Environment and Genes Related to Diseases, Xi’an Jiaotong University, Ministry of Education, Xi’an, China

**Keywords:** all-cause mortality, low muscle mass, neutrophil percentage-to-albumin ratio, NHANES, nutritional status, rheumatoid arthritis, risk stratification, systemic inflammation

## Abstract

**Introduction:**

Sarcopenia is an important extra-articular complication of rheumatoid arthritis (RA), caused by chronic inflammation, nutritional deficiency, and metabolic changes. Muscle mass is a key diagnostic indicator. The neutrophil percentage to albumin ratio (NPAR) is a readily available biomarker calculated from neutrophil percentage and serum albumin; however, its relationship with muscle mass reduction and prognosis in RA patients requires further clarification.

**Methods:**

The analysis was conducted on a cohort of 659 patients with RA who were admitted to hospital between Jan 2021 and Dec 2025. Low muscle mass (LMM) was defined using the 2019 Asian Working Group for Sarcopenia (AWGS) criteria with dual energy X-ray absorptiometry. The NPAR was calculated as neutrophil percentage divided by serum albumin. We performed multivariable logistic regression, restricted cubic spline (RCS) analysis, sensitivity analysis, receiver operating characteristic analysis, and subgroup analyses, with parallel analysis using 375 RA participants from the NHANES. Cox proportional hazards models assessed the association between NPAR and all-cause mortality in RA patients with LMM.

**Results:**

Among 167 hospitalized RA patients with LMM, elevated NPAR was independently associated with LMM risk after adjusting for confounders, with consistent sensitivity analyses. RCS analysis revealed a positive linear relationship. Patients with high NPAR showed significantly higher LMM prevalence than those with low NPAR. NPAR demonstrated strong discriminatory ability (AUC = 0.80, optimal cutoff = 9.55). In the NHANES cohort, elevated NPAR remained independently associated with LMM, with participants in the highest NPAR quartile substantially more likely to have LMM than those in the lowest quartile. Additionally, higher NPAR was associated with increased all-cause mortality in RA patients with LMM during follow-up.

**Conclusion:**

Higher NPAR is independently associated with LMM risk—a central diagnostic component of sarcopenia—in patients with RA, and may also predict worse survival among those with reduced muscle mass. These findings suggest that NPAR could facilitate early risk stratification and prognosis assessment for sarcopenia in RA clinical practice.

## 1 Introduction

Rheumatoid arthritis (RA) is a chronic systemic autoimmune disease characterized by persistent synovial inflammation, progressive joint destruction, and a wide range of extra-articular manifestations (1, 2). Among these, sarcopenia, defined as a progressive and generalized loss of skeletal muscle mass and function, has emerged as a prevalent yet underrecognized complication in patients with RA (3, 4). Previous studies suggest that approximately 20% to 40% of individuals with RA may have sarcopenia, and sarcopenia is associated with greater disability, lower quality of life, and higher mortality risk (5–7). The development of sarcopenia in RA is multifactorial, involving chronic systemic inflammation, decreased physical activity, malnutrition, and long-term exposure to glucocorticoids (3, 5).

**Figure 1:**
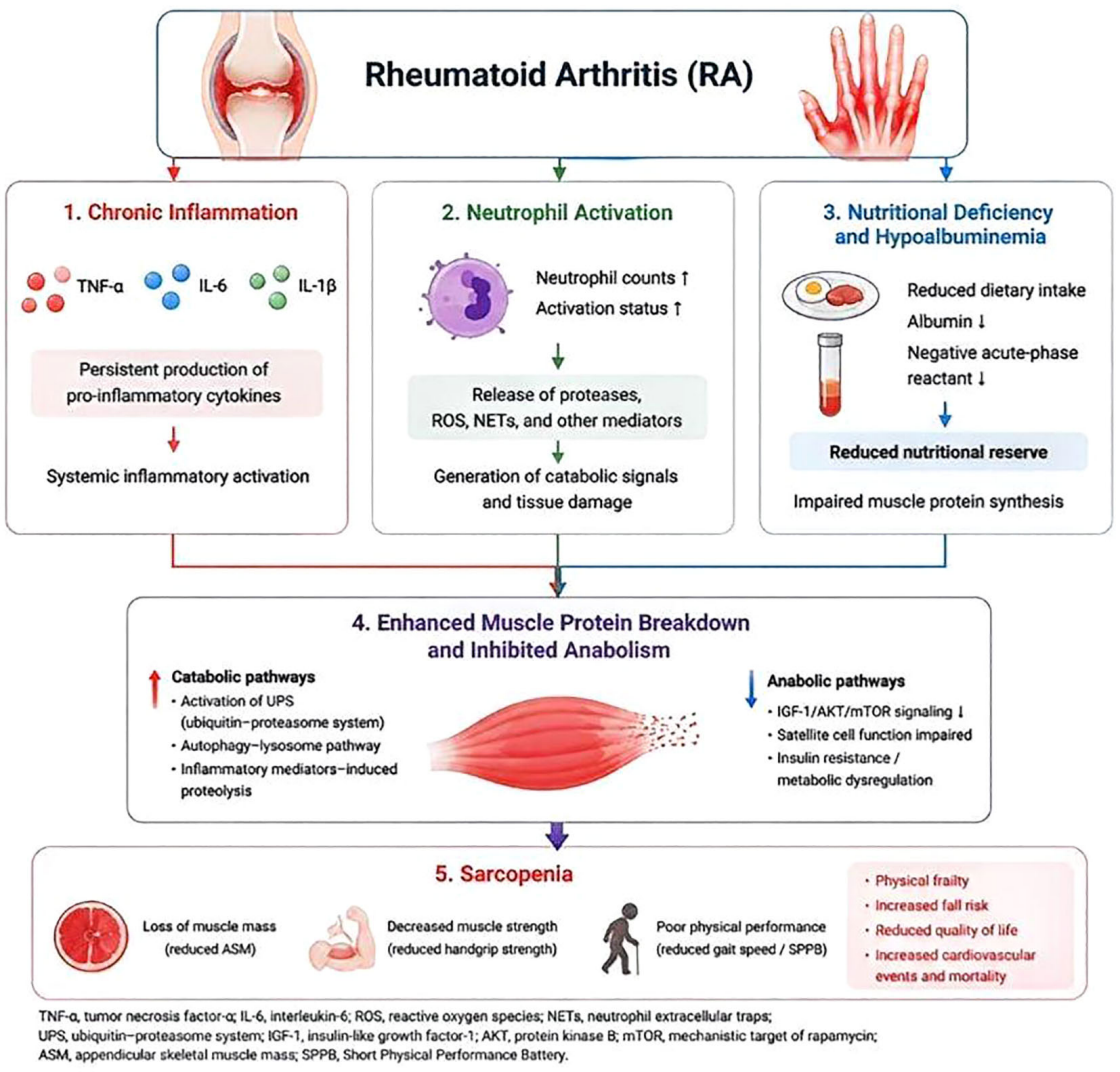
Proposed inflammatory–nutritional mechanism linking rheumatoid arthritis to sarcopenia.

Chronic inflammation plays a central role in the pathogenesis of both RA and sarcopenia (8). Pro-inflammatory cytokines such as tumor necrosis factor-alpha (TNF-α), interleukin-6 (IL-6), and interleukin-1β (IL-1β) not only contribute to joint destruction but also promote muscle protein breakdown and inhibit muscle synthesis (9, 10) (**Figure 1**). In addition, individuals with RA often have nutritional deficits, including hypoalbuminemia, low body mass index (BMI), and anemia, which may aggravate muscle wasting (11, 12). These findings highlight that both inflammatory burden and nutritional status are critical determinants in the development of sarcopenia among RA patients.

In recent years, integrated biomarkers such as the neutrophil percentage to albumin ratio (NPAR) have garnered considerable attention due to their clinical application value (13). NPAR has been demonstrated to be closely associated with increased risk of rheumatoid arthritis, chronic kidney disease, and their prognosis (14, 15). Similarly, composite indicators such as the neutrophil-to-lymphocyte ratio (NLR) and systemic immune-inflammation index (SII) have been found to be associated with muscle mass loss and functional decline (16, 17). However, research specifically investigating the relationship between NPAR and low muscle mass in RA patients remains limited. Previous studies have largely focused on demographic characteristics, disease activity, or single inflammatory markers (18–20), with limited attention paid to composite biomarkers (NPAR). Since RA patients are frequently accompanied by abnormal inflammation and metabolic abnormalities, integrated biomarkers can provide a more comprehensive evaluation of sarcopenia risk. Therefore, this study aims to investigate the relationship between NPAR and low muscle mass in RA patients and its prognostic implications.

In this study, we analyzed the association between NPAR and low muscle mass in a hospital-based RA cohort and observed the findings among RA participants from the US NHANES. We further examined whether NPAR is associated with all-cause mortality among RA patients with low muscle mass. We hypothesized that elevated NPAR would be associated with increased low muscle mass risk and worse survival outcomes, providing a simple, accessible biomarker for clinical risk stratification for sarcopenia in RA.

## 2 Materials and methods

### 2.1 Study design and participants

This cross-sectional study analyzed clinical data from patients with RA admitted to the Department of Rheumatology at the Second Affiliated Hospital of Zhejiang Chinese Medical University between January 2021 and December 2025. The study protocol was approved by the Medical Ethics Committee of the Second Affiliated Hospital of Zhejiang Chinese Medical University (Approval No. 2026-Research-094-01).

During the study period, a total of 1,356 hospitalizations with a primary diagnosis of RA were identified. For patients with multiple admissions, only data from the first hospitalization within the study period were included.

The inclusion criteria were as follows: (1) fulfillment of the 2010 American College of Rheumatology/European League Against Rheumatism (ACR/EULAR) classification criteria for RA; (2) age ≥18 years. The exclusion criteria included: (1) incomplete data for sarcopenia assessment; (2) missing NPAR values; (3) presence of malignancy; (4) coexistence of other autoimmune diseases; (5) pregnancy.

**Figure 2:**
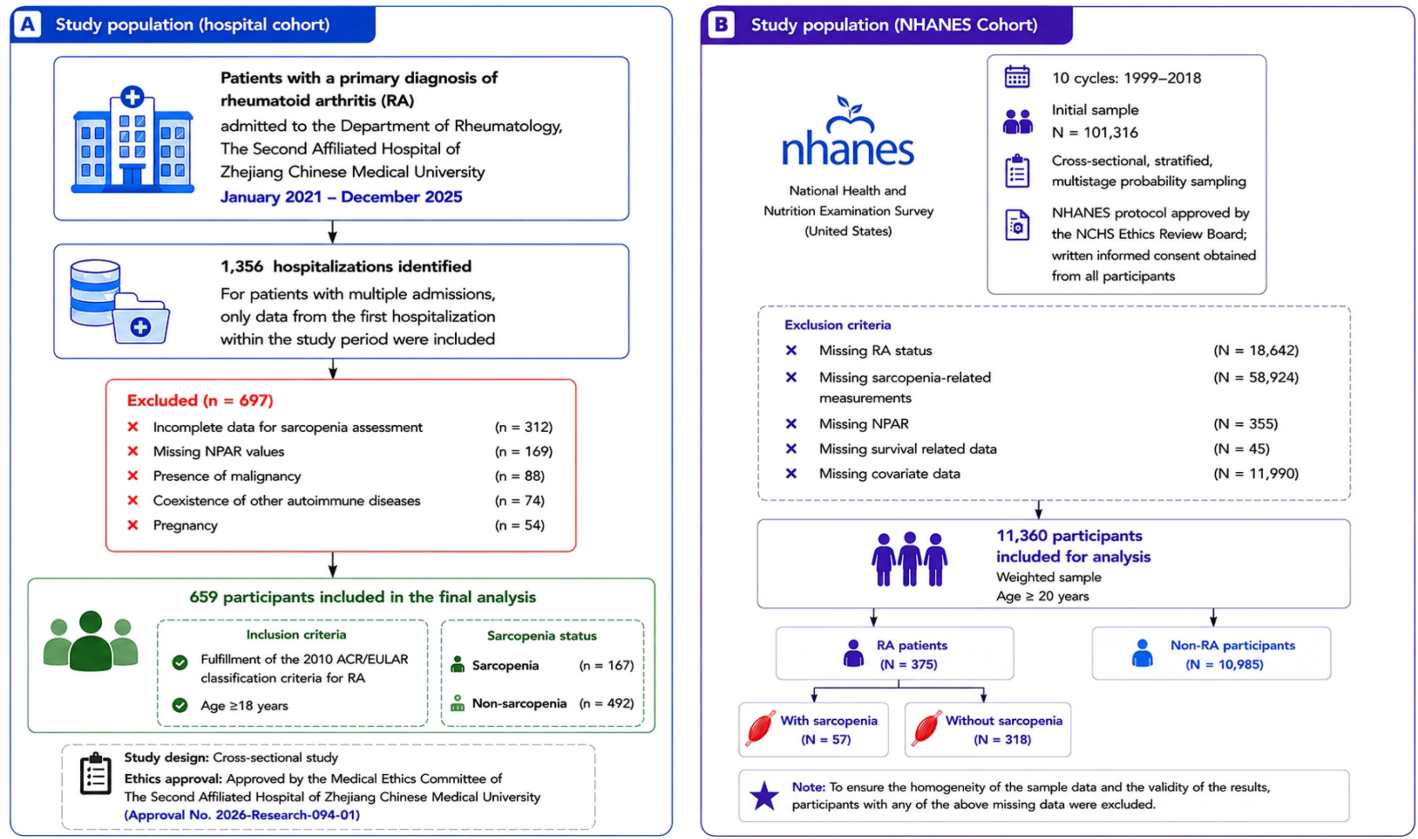
Flowchart of participant selection in the hospital cohort **(A)** and NHANES cohort **(B)**.

After applying these criteria, a total of 659 participants were included in the final analysis (**Figure 2A**).

### 2.2 Low muscle mass assessment

Low muscle mass was defined using the AWGS 2019 criteria based on appendicular skeletal muscle mass measured by dual-energy X-ray absorptiometry (DXA). The appendicular skeletal muscle index (ASMI) was calculated as appendicular skeletal muscle mass (kg) divided by height squared (m²), with cutoff values of <7.0 kg/m² for men and <5.4 kg/m² for women. All DXA scans were performed by trained technicians following manufacturer guidelines to ensure consistency.

### 2.3 Neutrophil percentage-to-albumin ratio

NPAR was calculated as the ratio of peripheral blood neutrophil percentage to serum albumin (g/dL). Blood samples were collected under fasting conditions, processed using standardized hematology analyzers, and albumin measured via the bromocresol green method. For descriptive purposes, participants were categorized into two groups based on the median NPAR value (9.64): Low-NPAR (<9.64) and High-NPAR (≥9.64). NPAR was primarily analyzed as a continuous variable in regression models.

### 2.4 Covariates

Demographic variables included age, sex, body mass index (BMI), smoking status, and alcohol consumption. Disease duration was categorized as 1–5 years, 6–10 years, 11–20 years, and >20 years. Comorbidities considered were diabetes, hypertension, cardiovascular diseases (CVDs), interstitial lung disease (ILD), chronic kidney disease (CKD), dyslipidemia, osteoporosis, and stroke. Laboratory parameters collected at admission included white blood cell (WBC) count, red blood cell (RBC) count, platelet (PLT) count, neutrophil (NEU) count, NEU percentage, monocyte (MON) count, lymphocyte (LYM) count, D-dimer (DDi), albumin (ALB), total cholesterol (TC), low-density lipoprotein cholesterol (LDL), high-density lipoprotein cholesterol (HDL), C-reactive protein (CRP), erythrocyte sedimentation rate (ESR), anti-citrullinated protein antibody (ACPA), rheumatoid factor (RF), and other derived inflammatory indices (SII, NLR, AISI, PLR, SIRI). The calculation formula for other inflammatory indicators is as follows: SII=(NEU*PLT)/LYM; NLR=NEU/LYM; AISI=(PLT*NEU*MON)/LYM; PLR=PLT/LYM; SIRI=MON*NEU/LYM.

### 2.5 Medication use

Data on the use of nonsteroidal anti-inflammatory drugs (NSAIDs), disease-modifying antirheumatic drugs (methotrexate, leflunomide, hydroxychloroquine, sulfasalazine, iguratimod), glucocorticoids, JAK inhibitors (tofacitinib, baricitinib) and TNF-α inhibitors were collected from electronic medical records.

### 2.6 Parallel observation cohort (NHANES)

Data from the U.S. National Health and Nutrition Examination Survey (NHANES, 1999–2018) were used for parallel analysis. A total of 375 participants were included in the final analysis (**Figure 2B**). RA participants were identified based on self-reported physician diagnosis. LMM was defined according to the European Working Group on Sarcopenia in Older People (EWGSOP) criteria, using appendicular skeletal muscle mass index (ASMI) thresholds of <7.26 kg/m² for men and <5.45 kg/m² for women. Mortality data were obtained from the National Death Index through December 31, 2019.

### 2.7 Statistical analysis

Continuous variables were summarized as mean ± standard deviation or median (interquartile range) and compared using t-tests or Mann–Whitney U tests, as appropriate. Categorical variables were expressed as counts and percentages and analyzed using chi-square tests. The association between NPAR and LMM was evaluated using multivariable logistic regression models with progressive adjustment: Model 1: unadjusted. Model 2: adjusted for age, sex, BMI, disease duration, smoking status, and alcohol consumption. Model 3: further adjusted for comorbidities, medication use, and laboratory parameters after excluding collinear factors with variance inflation factor (VIF) >5. To avoid over-adjustment, fully adjusted models did not include ALB and NEU percentage. Through receiver operating characteristic (ROC) curve analysis, the discriminatory ability of NPAR was compared with other inflammatory biomarkers including CRP, ESR, NLR, and SII. The area under the curve (AUC) was used for assessment, and pairwise comparisons were performed using the DeLong test. Restricted cubic spline (RCS) with four knots assessed potential nonlinear relationships. Subgroup analyses stratified by age, sex, BMI, disease duration, smoking status, and alcohol consumption were performed. In NHANES, we employed weighted logistic regression analysis and Cox proportional hazards models to assess the association between NPAR and all-cause mortality in rheumatoid arthritis patients with concomitant LMM. To reduce small sample bias and prevent overfitting, we applied Firth penalized maximum likelihood estimation in both the Logit model and Cox proportional hazards model.

Additionally, sensitivity analysis was performed to exclude the effects of patients with clinical infection or significantly elevated CRP (>100 mg/L) at admission. Data processing and analysis were performed using R version 4.3.3, SPSS version 20.0., along with Zstats 1.0 (www.zstats.net), with *P* values < 0.05 considered statistically significant.

## 3 Results

### 3.1 Baseline characteristics

Among the 659 RA patients in the hospital cohort, the mean age was 48.95 ± 18.02 years, and 63.73% were female, with a higher proportion in the low-NPAR group (68.09% vs. 59.39%, *P* = 0.020). The median disease duration was 9.0 years. Age distribution differed significantly between the low- and high-NPAR groups (*P* < 0.001), and the low-NPAR group had a higher proportion of patients aged 40–59 years (41.46% vs. 30.79%). The high-NPAR group had higher NEU count, NEU percentage, SII, NLR, AISI, and SIRI values, as well as greater glucocorticoid use. Furthermore, compared to the low-NPAR group, the high-NPAR group exhibited a notably higher prevalence of comorbidities such as OP and ILD (*P* < 0.05). Baseline characteristics stratified by NPAR median bisect are presented in **Table 1**.

**Table 1:** Comparison of baseline characteristics between RA patients in high and low NPAR groups.

Variables	Low-NPAR (n=329)	High-NPAR (n=330)	Overall (n=659)	P value
Demographics				
Gender, n (%)				0.020
Male	105 (31.91)	134 (40.61)	239 (36.27)	
Female	224 (68.09)	196 (59.39)	420 (63.73)	
Age (years), n (%)				<0.001
≤39	50 (15.24)	72 (21.95)	122 (18.60)	
40˜59	136 (41.46)	101 (30.79)	237 (36.13)	
60˜79	135 (41.16)	110 (33.54)	245 (37.35)	
≥80	7 (2.13)	45 (13.72)	52 (7.93)	
BMI (kg/m^2^), n (%)				0.407
<18.5	12 (3.65)	20 (6.06)	32 (4.86)	
18.5˜24.9	202 (61.40)	208 (63.03)	410 (62.22)	
25˜29.9	104 (31.61)	93 (28.18)	197 (29.89)	
≥30	11 (3.34)	9 (2.73)	20 (3.03)	
Smoking, n (%)				0.757
No	309 (93.92)	308 (93.33)	617 (93.63)	
Yes	20 (6.08)	22 (6.67)	42 (6.37)	
Drinking, n (%)				0.781
No	304 (92.40)	303 (91.82)	607 (92.11)	
Yes	25 (7.60)	27 (8.18)	52 (7.89)	
Disease duration (years), n (%)				
1˜5	117 (35.56)	108 (32.73)	225 (34.14)	0.167
6˜10	109 (33.13)	129 (39.09)	238 (36.12)	
11˜20	85 (25.84)	68 (20.61)	153 (23.22)	
>20	18 (5.47)	25 (7.58)	43 (6.53)	
Comorbidities				
Diabetes, n (%)	45 (13.68)	51 (15.45)	96 (14.57)	0.518
Hypertension, n (%)	73 (22.19)	93 (28.18)	166 (25.19)	0.076
CVDs, n (%)	62 (18.84)	58 (17.58)	120 (18.21)	0.673
CKD, n (%)	55 (16.72)	46 (13.94)	101 (15.33)	0.322
ILD, n (%)	12 (3.64)	26 (7.90)	38 (5.77)	0.019
Dyslipidemia, n (%)	53 (16.16)	61 (18.77)	114 (17.46)	0.380
Osteoporosis, n (%)	77 (23.40)	141 (42.70)	218 (33.08)	<0.001
Stroke, n (%)	39 (11.85)	46 (13.94)	85 (12.90)	0.425
Low muscle mass, n (%)	49 (14.89)	118 (35.76)	167 (25.34)	<0.001
Laboratory examination				
RF (IU/mL), n (%)				0.124
≤20	58 (17.63)	74 (22.42)	132 (20.03)	
>20	271 (82.37)	256 (77.58)	527 (79.97)	
ACPA (U/mL), n (%)				0.713
0-5	38 (11.55)	43 (13.03)	81 (12.29)	
5.1~200	148 (44.98)	139 (42.12)	287 (43.55)	
>200	143 (43.47)	148 (44.85)	291 (44.16)	
CRP (mg/L), n (%)				0.343
0-8	62 (18.84)	72 (21.82)	134 (20.33)	
>8	267 (81.16)	258 (78.18)	525 (79.67)	
ESR (mm/h), n (%)				0.425
Normal	290 (88.15)	284 (86.06)	574 (87.10)	
Higher	39 (11.85)	46 (13.94)	85 (12.90)	
WBC (x10^9^/L), Mean ± SD	6.19 ± 2.07	5.99 ± 1.91	6.09 ± 1.99	0.198
RBC (x10^12^/L), Mean ± SD	4.05 ± 0.68	3.97 ± 0.65	4.01 ± 0.67	0.136
PLT (x10^9^/L), Mean ± SD	206.06 ± 69.83	206.44 ± 69.05	206.25 ± 69.39	0.945
NEU (x10^9^/L), Mean ± SD	2.86 ± 0.68	5.39 ± 2.00	4.12 ± 1.96	<0.001
NEU percentage, Mean ± SD	28.62 ± 6.80	53.97 ± 19.99	41.31 ± 19.59	<0.001
MONO (x10^9^/L), Mean ± SD	0.42 ± 0.16	0.42 ± 0.15	0.42 ± 0.15	0.990
LYM (x10^9^/L), Mean ± SD	1.57 ± 0.68	1.63 ± 0.72	1.60 ± 0.71	0.250
DDi (mg/L), Mean ± SD	1259.19 ± 730.65	1320.42 ± 701.80	1289.85 ± 716.46	0.273
TC (mmol/L), Mean ± SD	4.01 ± 1.11	3.93 ± 1.10	3.97 ± 1.11	0.380
HDL (mmol/L), Mean ± SD	1.21 ± 0.40	1.15 ± 0.41	1.18 ± 0.41	0.079
LDL (mmol/L), Mean ± SD	2.27 ± 0.85	2.01 ± 0.79	2.14 ± 0.83	<0.001
ALB (g/dL), Mean ± SD	3.99 ± 0.40	3.75 ± 0.42	3.87 ± 0.43	<0.001
Inflammation index				
SII, Mean ± SD	432.54 ± 260.07	825.97 ± 687.22	629.56 ± 555.52	<0.001
NLR, Mean ± SD	2.19 ± 1.26	4.53 ± 8.60	3.37 ± 6.25	<0.001
AISI, Mean ± SD	147.27 ± 108.77	286.11 ± 223.80	216.79 ± 189.13	<0.001
PLR, Mean ± SD	151.38 ± 80.54	154.87 ± 117.13	153.13 ± 100.48	0.656
SIRI, Mean ± SD	0.75 ± 0.57	1.52 ± 1.60	1.13 ± 1.26	<0.001
Medication use				
NSAIDs, n (%)	256 (77.81)	262 (79.39)	518 (78.60)	0.620
Glucocorticoids, n (%)	80 (24.32)	134 (40.60)	214 (32.47)	<0.001
Methotrexate, n (%)	164 (49.85)	165 (50.00)	329 (49.92)	0.969
Leflunomide, n (%)	56 (17.02)	56 (16.97)	112 (17.00)	0.986
Hydroxychloroquine, n (%)	36 (10.94)	27 (8.18)	63 (9.56)	0.228
Sulfasalazine, n (%)	2 (0.61)	2 (0.61)	4 (0.61)	1.000
Iguratimod, n (%)	58 (17.63)	58 (17.58)	116 (17.60)	0.986
Tofacitinib, n (%)	66 (20.06)	74 (22.42)	140 (21.24)	0.458
Baricitinib, n (%)	3 (0.91)	12 (3.64)	15 (2.28)	0.019
TNF-α inhibitor, n (%)	55 (16.72)	36 (10.91)	91 (13.81)	0.031

### 3.2 Univariate and multivariate logistic regression analyses

Univariate logistic regression analysis identified smoking, disease duration, osteoporosis, GCs, RF, NPAR, ALB, NEU, NEU percentage, SII, NLR, AISI and SIRI as variables significantly associated with the presence of LMM (*P* < 0.05). Variables with p < 0.05 in the univariate analysis were subsequently entered into the multivariate logistic regression model using backward stepwise elimination. Multivariate analysis demonstrated that the NPAR were independent risk factors for LMM. For every 1-unit increase in NPAR, the risk increased by 26% (OR = 1.26, 95%CI: 1.01 ~ 2.87, *P* = 0.003); **Supplementary Table 1** presents the full results of the regression.

### 3.3 Association between NPAR and sarcopenia

**Figure 3:**
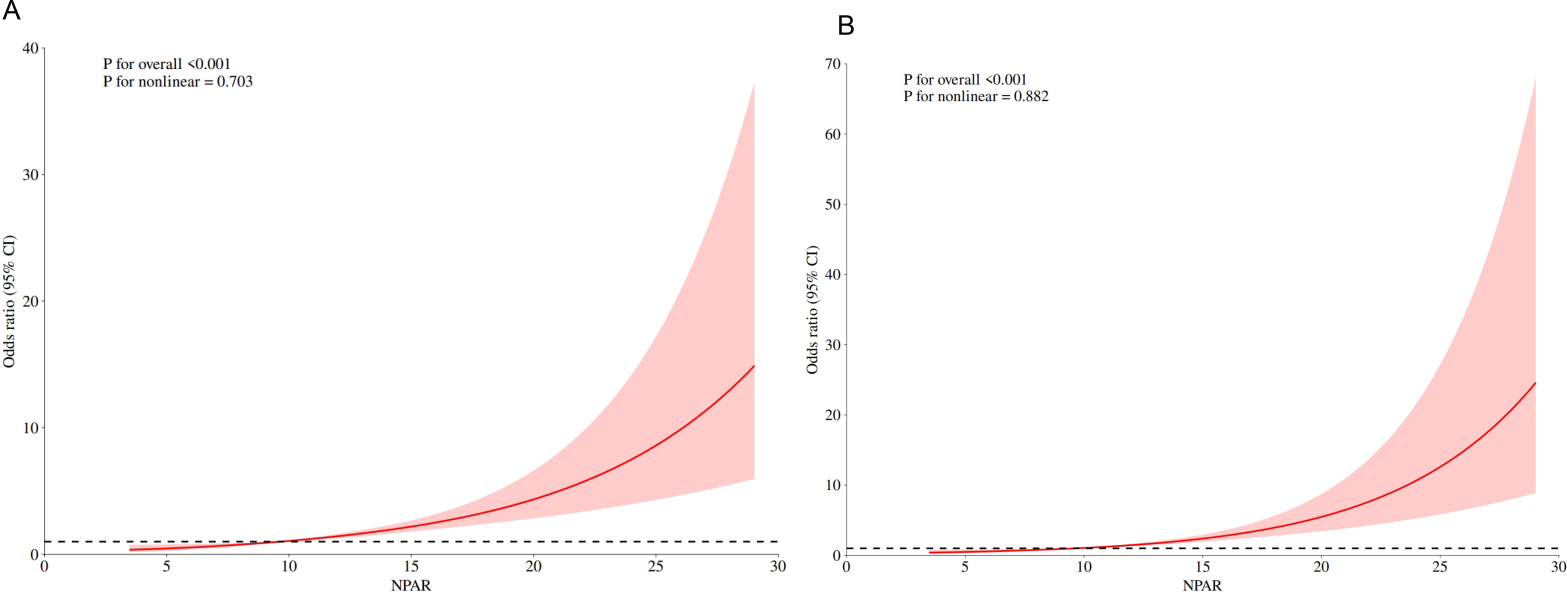
Restricted cubic spline analysis of the association between NPAR and low muscle mass risk in the hospital RA cohort. **(A)** unadjusted RCS, **(B)** covariate-adjusted RCS.

After eliminating multicollinearity among confounding factors (**Supplementary Table 2**), the association remained statistically significant even after adjustment for all covariates (**Table 2**) (OR = 1.20; 95% CI: 1.09–1.31; P<0.001). Whether stratified by median (OR = 2.98; 95%CI: 2.01–4.42) or by cutoff value (OR = 4.57; 95%CI: 2.63–7.92), the high NPAR group demonstrated significantly elevated prevalence risk of LMM compared with the low NPAR group (all P<0.001). Additionally, RCS analysis, adjusted for relevant covariates, revealed a significant linear positive relationship between NPAR levels and LMM prevalence, as shown in **Figure 3** (P for trend < 0.001), indicating that higher NPAR levels are associated with an elevated LMM risk.

**Table 2:** Multimodel logistic regression analysis of the association between NPAR and low muscle mass risk in patients with RA.

Variables	Model 1		Model 2		Model 3	
	OR (95% CI)	P	OR (95% CI)	P	OR(95% CI)	P
NPAR (Total)	1.19 (1.15~1.24)	<0.001	1.25 (1.19~1.33)	<0.001	1.21(1.13~1.29)	<0.001
NPAR group by median						
Low-NPAR	Ref.		Ref.		Ref.	
High-NPAR	2.99(2.02~4.41)	<0.001	3.87 (2.36~6.34)	<0.001	2.98(2.01~4.42)	<0.001
P for Trend		<0.001		<0.001		<0.001
NPAR group by cut-off						
Low-NPAR	Ref.		Ref.		Ref.	
High-NPAR	3.43 (2.35~5.02)	<0.001	4.60 (2.67~7.69)	<0.001	4.57(2.63~7.92)	<0.001
P for Trend		<0.001		<0.001		<0.001

OR, odds ratio; CI, confidence interval.

Model 1: unadjusted.

Model 2: adjusted for age, sex, BMI, disease duration, smoking status, and alcohol consumption.

Model 3: further adjusted for comorbidities, medication use, and laboratory indicators, excluding VIF>5, albumin and neutrophil percentage to avoid bias.

### 3.4 Predictive performance of NPAR

**Figure 4:**
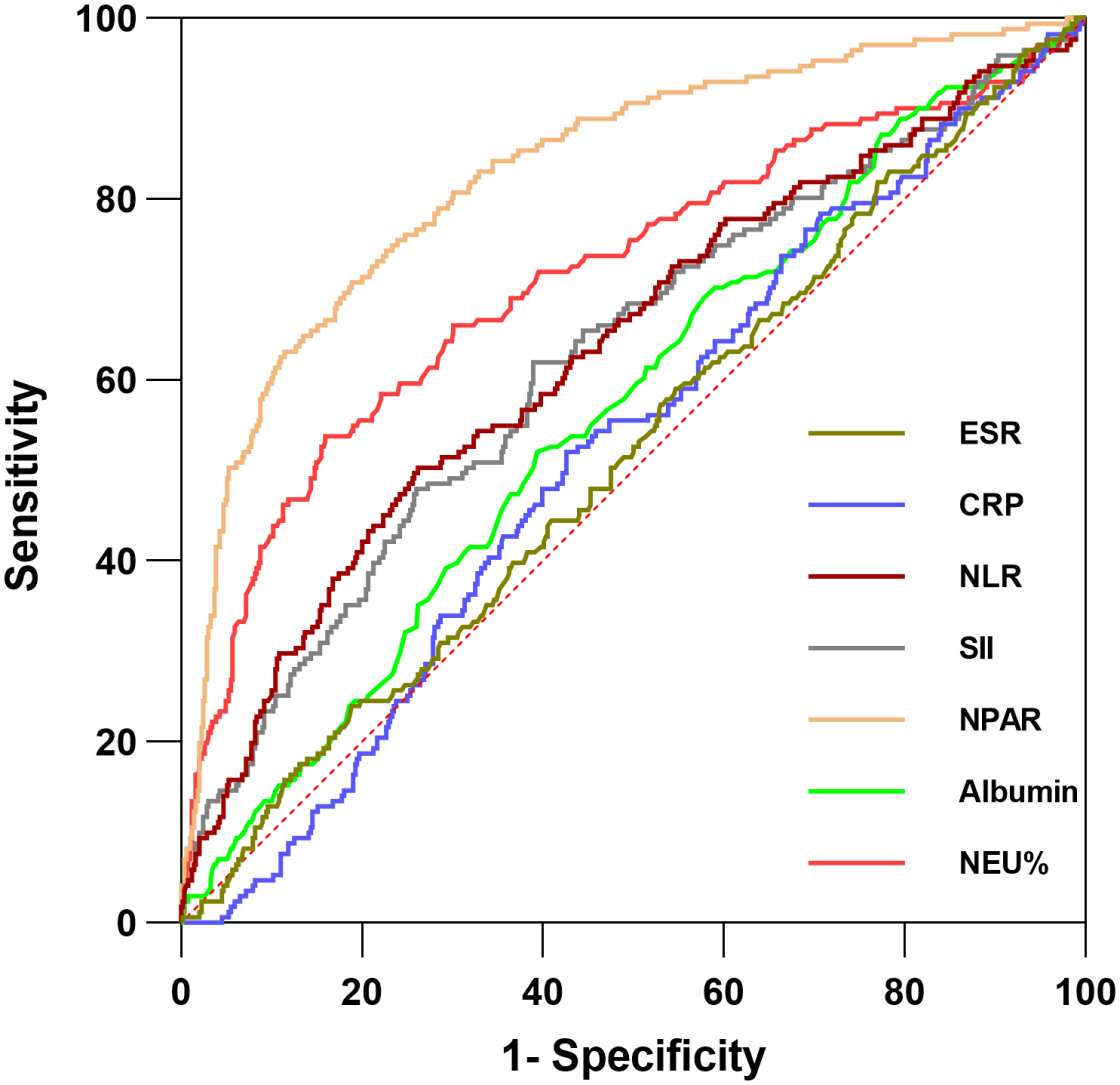
Receiver operating characteristic curve analysis of NPAR, its components, and other inflammatory markers for predicting LMM in the hospital RA cohort.

To evaluate NPAR’s incremental value, we compared its discriminatory performance with that of its individual components (neutrophil percentage and albumin) and other widely used inflammatory markers (CRP, ESR, NLR, and SII). As shown in **Figure 4** and **Supplementary Table 3**, NPAR achieved the highest AUC (0.80; 95% CI: 0.76–0.84), significantly outperforming neutrophil percentage (AUC = 0.69), albumin (AUC = 0.57), CRP (AUC = 0.52), ESR (AUC = 0.52), NLR (AUC = 0.63), and SII (AUC = 0.62), with all pairwise comparisons significant (P < 0.001) by DeLong’s test. The optimal NPAR cutoff determined by the maximum Youden index was 9.55, yielding sensitivity of 72.0% and specificity of 76.5%. Overall, these findings suggest that NPAR may be more suitable as an adjunct for risk stratification than as a stand-alone diagnostic tool.

### 3.5 Subgroup analyses

**Figure 5:**
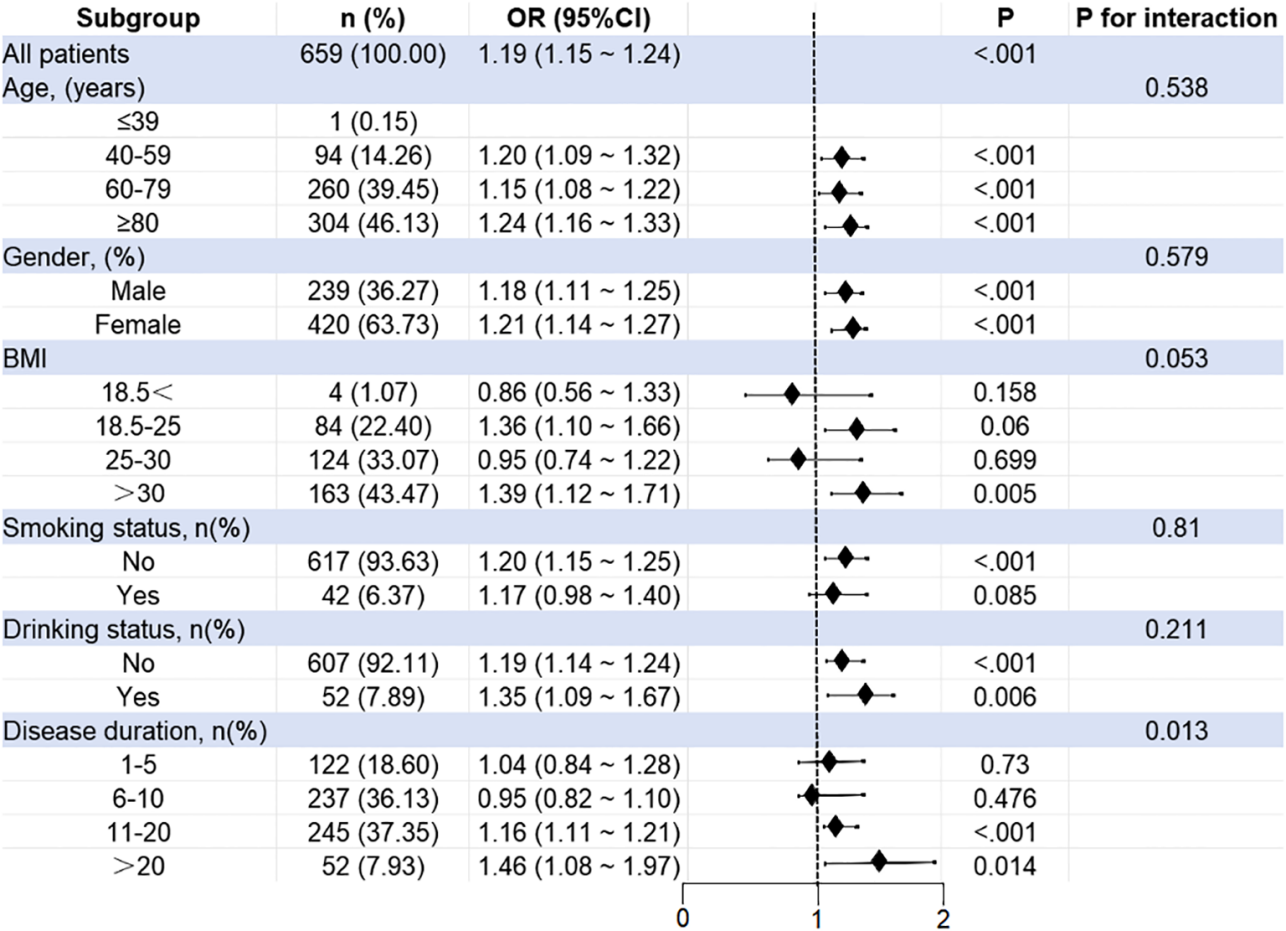
Subgroup and interaction analyses of the association between NPAR and LMM risk in the hospital RA cohort.

The association between NPAR and LMM persisted across gender, age, BMI, disease duration, smoking status, and alcohol consumption subgroups. Notably, patients with disease duration 11–20 year (OR 1.16, 95% CI (1.11 ~ 1.21), and >20 years (OR 1.46, 95% CI 1.08–1.97) showed stronger associations, suggesting that in cases of elevated NPAR levels, a longer duration of RA may significantly increase the risk of LMM (**Figure 5**).

### 3.6 Sensitivity analysis

To perform sensitivity analysis excluding the confounding effects of infection or acute inflammation, we excluded patients with clinical infection or elevated C-reactive protein (> 100mg/L). A total of 246 patients were excluded, leaving 413 patients for analysis. The results demonstrated (**Supplementary Table 4**) that the positive correlation between NPAR and LMM remained stable and even strengthened. After comprehensive adjustment, each unit increase in NPAR was associated with a 31% increase in LMM risk (OR = 1.31, 95% CI: 1.18-1.45, P<0.001); the high NPAR group had a nearly 9-fold increased risk compared with the low NPAR group (OR = 8.76, 95% CI: 3.31-23.20, P<0.001).

### 3.7 Parallel analysis in NHANES RA participants

The study included 375 participants with complete data (202 females, 173 males). **Table 3** shows the weighted characteristics categorized by NPAR levels, including demographics, lifestyle factors, chronic disease history, and inflammation indices. Weighted logistic regression analysis demonstrated a significant positive correlation between NPAR levels and LMM, which remained statistically significant even after adjustment for all confounding factors (OR = 1.28; 95% CI: 1.09-1.49; P = 0.003) (**Table 4**). When stratified by quartiles, the risk of LMM in the highest quartile (Q4) was significantly elevated compared to the lowest quartile (Q1) (OR = 4.88; 95% CI: 1.63-14.61; P = 0.007). Weighted Firth penalized logistic regression analysis further confirmed this association after full adjustment (OR = 1.27; 95% CI: 1.08-1.48; P = 0.004), with participants in the Q4 group showing significantly higher risk compared to the Q1 group (OR = 4.62; 95% CI: 1.56-13.68; P = 0.006) (**Supplementary Table 5**).

**Table 3:** Comparison of baseline characteristics between RA patients in high and low NPAR groups in NHANES.

Variable	Total (n = 375)	Low-NPAR (n=166)	High-NPAR (n=209)	Statistic	P
Age, (year)				χ²=0.61	0.786
20-45	118 (37.18)	49 (35.46)	69 (38.59)		
46-65	188 (51.97)	89 (54.20)	99 (50.14)		
>65	69 (10.85)	28 (10.34)	41 (11.27)		
Gender, (%)				χ²=5.43	0.057
Male	173 (49.40)	85 (56.07)	88 (43.97)		
Female	202 (50.60)	81 (43.93)	121 (56.03)		
Race, (%)				χ²=16.39	<0.001
Non-Hispanic White	59 (7.02)	17 (3.45)	42 (9.93)		
Non-Hispanic Black	178 (69.17)	76 (65.27)	102 (72.36)		
Mexican American	91 (12.03)	46 (13.40)	45 (10.91)		
Other Races	47 (11.78)	27 (17.89)	20 (6.80)		
BMI				χ²=11.62	0.083
18.5<	4 (1.30)	3 (0.86)	1 (1.65)		
18.5-25	84 (25.46)	37 (22.67)	47 (27.74)		
25-30	124 (31.89)	67 (40.89)	57 (24.55)		
>30	163 (41.35)	59 (35.58)	104 (46.05)		
Education, (%)				χ²=2.78	0.447
Below high school	82 (15.01)	38 (18.40)	44 (12.25)		
High school	101 (28.49)	39 (26.96)	62 (29.74)		
Above high school	192 (56.50)	89 (54.64)	103 (58.01)		
Marital status, (%)				χ²=3.02	0.292
Married	219 (64.28)	102 (66.62)	117 (62.37)		
Widowed/Divorced	123 (28.25)	50 (24.29)	73 (31.47)		
Never married	33 (7.47)	14 (9.09)	19 (6.16)		
PIR, n (%)				χ²=1.49	0.641
<1.3	113 (23.39)	46 (21.07)	67 (25.29)		
1.3-3.5	136 (33.08)	63 (32.21)	73 (33.79)		
≥3.5	126 (43.53)	57 (46.72)	69 (40.92)		
Smoking status, n(%)				χ²=4.47	0.228
Never	142 (32.89)	59 (27.32)	83 (37.43)		
Former	106 (30.84)	48 (34.37)	58 (27.97)		
Now	127 (36.27)	59 (38.32)	68 (34.60)		
Drinking status, n(%)				χ²=1.92	0.529
Mild drinking	186 (49.32)	85 (53.18)	101 (46.17)		
Moderate drinking	129 (33.83)	57 (31.86)	72 (35.43)		
Heavy drinking	60 (16.85)	24 (14.96)	36 (18.40)		
Physical activity, n(%)				χ²=1.53	0.363
Low physical activity	128 (32.55)	59 (29.24)	69 (35.24)		
High physical activity	247 (67.45)	107 (70.76)	140 (64.76)		
Hypertension, n(%)				χ²=4.49	0.100
No	175 (54.09)	73 (48.05)	102 (59.02)		
Yes	200 (45.91)	93 (51.95)	107 (40.98)		
Diabetes, n(%)				χ²=6.59	0.009
No	297 (86.37)	141 (91.40)	156 (82.26)		
Yes	78 (13.63)	25 (8.60)	53 (17.74)		
Hyperlipidemia				χ²=0.07	0.865
No	83 (20.93)	33 (20.30)	50 (21.45)		
Yes	292 (79.07)	133 (79.70)	159 (78.55)		
Sarcopenia				χ²=12.07	<0.001
No	318 (89.47)	151 (95.57)	167 (84.50)		
Yes	57 (10.53)	15 (4.43)	42 (15.50)		
Mortality, n(%)				χ²=1.83	0.198
No	302 (84.24)	138 (87.06)	164 (81.94)		
Yes	73 (15.76)	28 (12.94)	45 (18.06)		
Inflammation Index					
SII, Mean (SE)	593.75 (26.89)	374.80 (12.52)	772.31 (40.76)	t=9.73	<0.001
NLR, Mean (SE)	2.22 (0.07)	1.47 (0.03)	2.82 (0.10)	t=13.27	<0.001
AISI, Mean (SE)	353.68 (24.33)	208.81 (10.55)	471.83 (38.98)	t=6.74	<0.001
PLR, Mean (SE)	131.26 (3.32)	114.25 (4.24)	145.13 (4.32)	t=5.37	<0.001
SIRI, Mean (SE)	1.28 (0.06)	0.80 (0.03)	1.66 (0.09)	t=8.92	<0.001

**Table 4:** The association between NPAR level and low muscle mass risk in patients with RA.

	Model 1 OR (95% CI) P-value	Model 2 OR (95% CI) P-value	Model 3 OR (95% CI) P-value
NPAR (Total)	1.26 (1.11 ~ 1.42) <0.001	1.26 (1.10 ~ 1.45) <0.001	1.28 (1.09 ~ 1.49) 0.003
NPAR levels (Quantile)			
Q1	Ref.	Ref.	Ref.
Q2	1.23 (0.36 ~ 4.24) 0.739	0.81 (0.22 ~ 2.91) 0.744	0.66 (0.18 ~ 2.40) 0.525
Q3	2.47 (0.70 ~ 8.67) 0.162	2.10 (0.58 ~ 7.60) 0.262	1.26 (0.28 ~ 5.72) 0.769
Q4	5.97 (2.08 ~ 17.12) 0.001	4.89 (1.77 ~ 13.51) 0.003	4.88 (1.63 ~ 14.61) 0.007
P for trend	<0.001	<0.001	<0.001

Model 1: Not adjusted.

Model 2: Adjusted by age, gender, and race.

Model 3: Adjusted by age, Gender, BMI, Race, PIR, Education, Marital status, Smoking status, Drinking status, Physical activity, Diabetes, Hypertension, and Hyperlipidemia. P < 0.05 indicates statistical significance.

**Figure 6:**
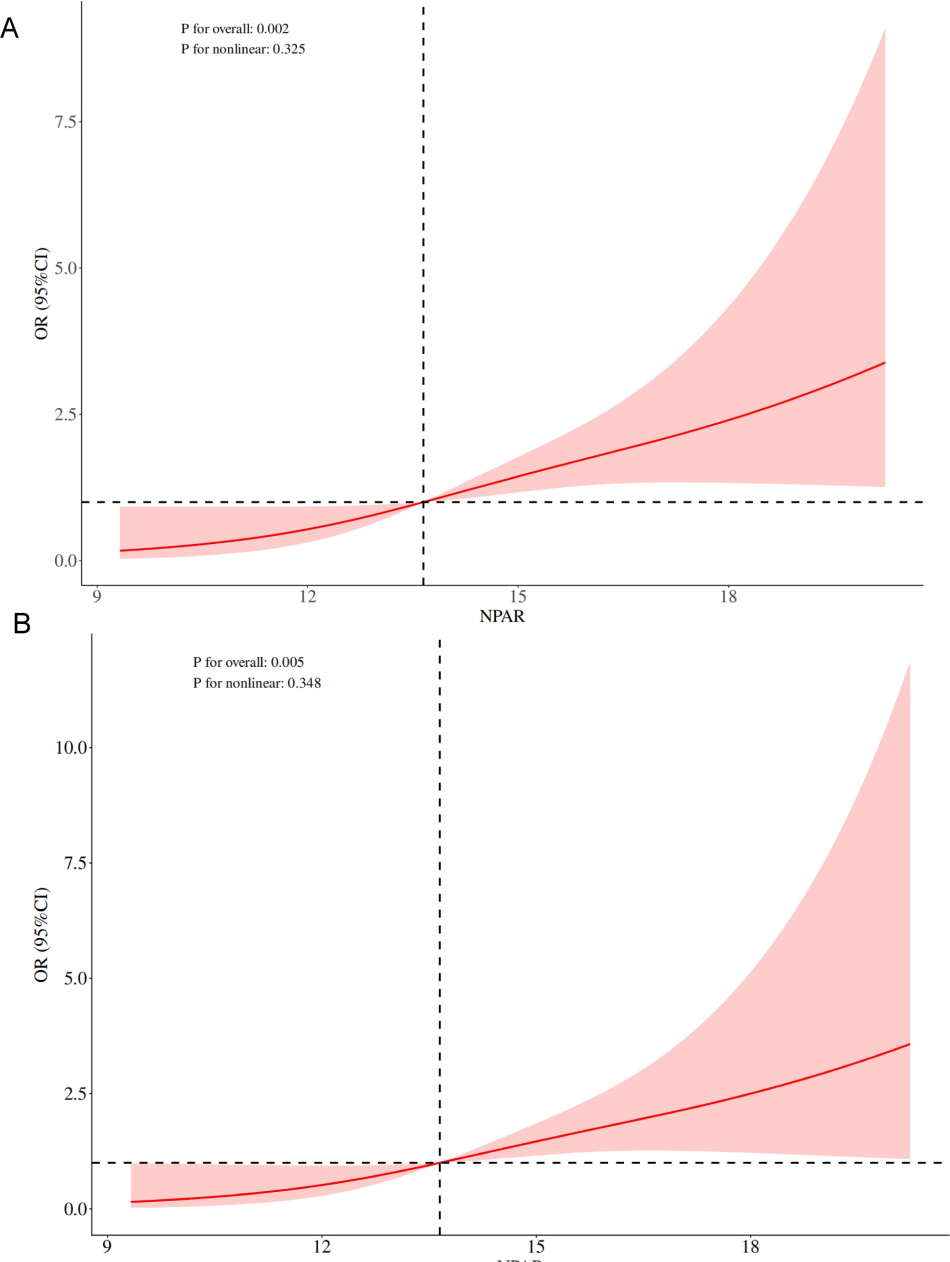
Restricted cubic spline analysis of the association between NPAR and low muscle mass risk in the NHANES validation cohort. **(A)** unadjusted RCS, **(B)** covariate-adjusted RCS.

**Figure 7:**
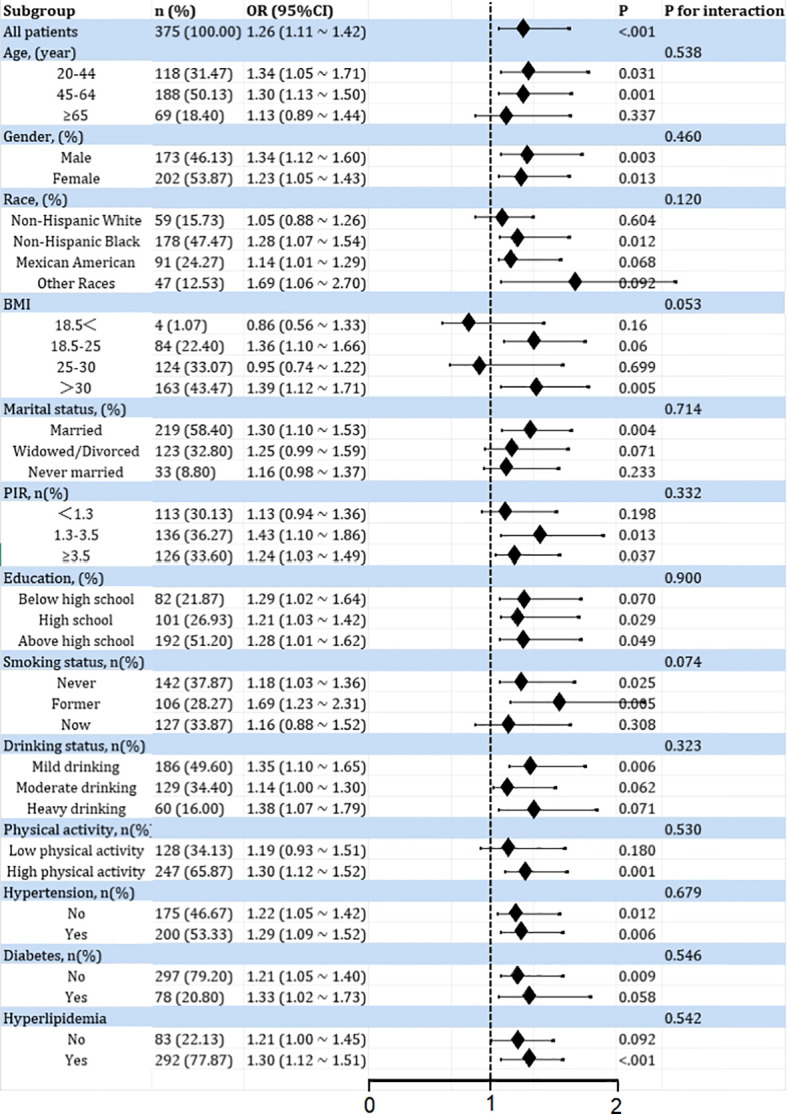
Subgroup and interaction analyses of the association between NPAR and low muscle mass risk in RA participants from the NHANES validation cohort.

Additionally, RCS analysis, adjusted for relevant covariates, revealed a significant linear positive relationship between NPAR levels and LMM prevalence, as shown in **Figure 6** (P = 0.005). Subgroup and interaction analyses were conducted to evaluate factors potentially modifying the relationship between NPAR levels and the risk of LMM. NPAR levels demonstrated a consistent and statistically significant positive association with LMM (**Figure 7**).

### 3.8 NPAR and all-cause mortality

During a median follow-up of 8.3 years, 21 deaths occurred among NHANES RA participants with LMM. Elevated NPAR was associated with increased all-cause mortality in Cox proportional hazards models after adjusting for all covariates (HR 1.12, 95% CI 1.02–1.22, P = 0.013) (**Table 5**). In the Firth penalized model, this association persisted after adjusting for age, sex, race, and BMI (HR 1.11, 95% CI 1.02–1.21, P = 0.018) (**Supplementary Table 5**). Kaplan–Meier curves stratified by NPAR tertiles illustrate lower survival in patients with higher NPAR (**Supplementary Figure 1**).

**Table 5:** The association of NPAR level with all-cause mortality among RA patients with low muscle mass.

	Model 1 HR (95% CI) P-value	Model 2 HR (95% CI) P-value	Model 3 HR (95% CI) P-value
NPAR (Total)	1.10 (1.02 - 1.18) 0.018	1.09 (1.01 - 1.18) 0.037	1.12 (1.02 - 1.22) 0.013
NPAR levels (Quantile)			
Q1	Ref.	Ref.	Ref.
Q2	2.04 (0.86 - 4.87) 0.107	2.11 (0.89 - 5.01) 0.090	1.87 (0.82 - 4.25) 0.134
Q3	1.77 (0.76 - 4.13) 0.184	1.74 (0.74 - 4.11) 0.207	1.50 (0.57 - 3.97) 0.416
Q4	2.74 (1.28 - 5.88) 0.010	2.74 (1.24 - 6.03) 0.012	2.40 (1.11 - 5.23) 0.027
P for trend	<0.001	<0.001	<0.001

Model 1: Not adjusted.

Model 2: Adjusted by age, gender, race.

Model 3: Age, Gender, BMI, Race, PIR, Education, Marital status, Smoking status, Drinking status, Physical activity, Diabetes, Hypertension, and Hyperlipidemia. P < 0.05 indicates statistical significance.

## 4 Discussion

RA is a systemic autoimmune disease, which causes persistent synovitis and progressive joint destruction often associated with significant disability and socioeconomic burden (21). Beyond articular manifestations, RA is increasingly recognized for its association with debilitating extra-articular comorbidities, such as sarcopenia, which significantly impair patients’ quality of life and prognosis (22, 23). Chronic inflammation and systemic features of RA lead to muscle catabolism. Moreover, the inflammation-related nutritional deficiency may further increase the risk of muscle wasting (24). Therefore, identifying simple, accessible biomarkers to screen for sarcopenia risk in RA is crucial for holistic patient management. This cross-sectional study, based on parallel analysis of a hospital cohort and the NHANES cohort, assessed the relationship between NPAR and the risk of LMM in patients with RA. Our key findings, validated in the NHANES cohort, demonstrate that elevated NPAR is an independent, linear risk factor for sarcopenia and is further associated with increased all-cause mortality in affected individuals.

RA is characterized by persistent activation of innate and adaptive immune pathways, resulting increased circulating levels of pro-inflammatory cytokines such as TNF-α and IL-6 (25–27). These cytokines enhance the breakdown of skeletal muscle proteins through both ubiquitin-proteasome and autophagy-mediated pathways while also impeding anabolic signaling. These processes contribute to a progressive decline in muscle mass and function (28, 29). Neutrophils, the principal effector cells of innate immunity, may promote tissue catabolism through the release of reactive oxygen species, proteases and inflammatory mediators (30, 31). Hypoalbuminemia may represent the negative acute-phase response and insufficient intake of protein, thereby worsening the catabolic stress on skeletal muscle (32). In our cohort, albumin alone showed limited discriminative ability for low muscle mass (AUC = 0.57), whereas neutrophil percentage demonstrated moderate performance (AUC = 0.69). NPAR’s superior performance (AUC = 0.80) suggests the ratio effectively amplifies the inflammatory signal of neutrophil percentage while normalizing inter-individual variation in metabolic status. This interpretation is consistent with the ASPEN position that serum albumin reflects inflammatory status rather than nutritional status per se (33). Furthermore, recent gene integration analyses have identified neutrophil extracellular trap formation as a key pathway shared between RA and sarcopenia, providing a molecular basis for the neutrophil-LMM association (34). The nutritional contribution of NPAR—while biologically plausible—remains hypothetical in this cohort and requires direct validation through dedicated nutritional assessments.

The subgroup analyses further strengthened the robustness of the association between NPAR and sarcopenia. The positive association persisted across major subgroups, including sex, age, BMI, disease duration, smoking status, and alcohol consumption. Notably, the association was especially pronounced in patients with longer disease duration, particularly among patients with disease duration of 11–20 years and >20 years. This finding is clinically meaningful because longer disease duration may reflect cumulative exposure to systemic inflammation, repeated flares of disease, functional limitations, glucocorticoid treatment and comorbidity burden, all of which can lead to progressive muscle loss (35). Therefore, in patients with long disease duration, elevated NPAR may indicate a particularly high-risk phenotype requiring closer monitoring of muscle mass, physical performance, and nutritional status.

Mechanistically, the link between elevated NPAR and sarcopenia may also involve immune-mediated modulation of muscle homeostasis (36). The proteolytic enzymes, reactive oxygen species, and pro-inflammatory mediators released by neutrophils can modulate satellite cell activity and myofiber regeneration (37). Additionally, hypoalbuminemia may reflect altered hepatic acute-phase response and systemic cytokine burden, which further contributes to metabolic dysregulation (38). These intertwined pathways highlight that NPAR may act as a proxy not just for inflammation and nutrition but also for immune-metabolic crosstalk that drives muscle catabolism.

Clinically, our findings extend beyond LMM risk. The association between high NPAR and increased all-cause mortality suggests that NPAR may have prognostic value, potentially reflecting the cumulative impact of chronic inflammation, nutritional status, and comorbidity burden. In the hospital cohort, NPAR demonstrated strong discriminatory ability for sarcopenia (AUC = 0.80; optimal cutoff = 9.55). Notably, although neither CRP nor ESR showed a significant association with LMM, this does not invalidate the role of inflammation in muscle loss. Rather, CRP and ESR primarily reflect hepatic acute-phase responses to systemic cytokines such as IL-6 (38), whereas neutrophils can directly promote tissue catabolism through the release of proteases and reactive oxygen species (30, 31). This mechanistic distinction may explain why NPAR—a neutrophil-based marker—captures muscle-relevant inflammatory signals that conventional acute-phase reactants miss. In sensitivity analyses excluding patients with infection or markedly elevated CRP, the NPAR–LMM association remained significant, but the effect estimate increased substantially with wider confidence intervals (OR = 8.76, 95% CI: 3.31–23.20). This should be interpreted with caution, as it may reflect both a genuine signal in a clinically cleaner subgroup and reduced statistical precision due to sample size reduction. Overall, these findings suggest that the NPAR–LMM association is not driven solely by acute inflammation, supporting its utility as a stable biomarker in clinically stable RA patients.

The strong and near-linear relationship between NPAR and LMM indicates its potential utility for early identification of at-risk RA patients. Clinically, patients with elevated NPAR may benefit from more integrated interventions, including targeted anti-inflammatory therapy to optimize disease-modifying antirheumatic drugs (DMARDs) or biologics to reduce systemic inflammation (39, 40). Nutritional support: protein supplementation or diet interventions to counter hypoalbuminemia and anabolic resistance (41, 42). Physical rehabilitation refers to structured exercise programs designed to maintain muscle mass and function (43, 44). Early identification using NPAR may therefore prevent progression of sarcopenia, reduce frailty, and improve long-term survival (45, 46).

This study has several strengths. First, it focused on a clinically important but often overlooked complication of RA. Second, diagnostic reliability was improved by DXA-based assessment of skeletal muscle mass in the hospital cohort. Third, the association between NPAR and LMM was evaluated via multivariable logistic regression, RCS analysis, ROC analysis, and subgroup analyses. Fourth, parallel analysis of the findings was carried out using an independent NHANES cohort, and a mortality analysis further extended the clinical relevance of NPAR.

Nevertheless, several limitations should be acknowledged. First, the cross-sectional design of the hospital cohort precludes causal inference. Although elevated NPAR was associated with LMM, it remains unclear whether increased NPAR contributes to muscle loss or reflects the inflammatory and nutritional consequences of sarcopenia. Second, residual confounding cannot be fully excluded. Specifically, we lack detailed data on disease activity scores (such as DAS28) and cumulative glucocorticoid exposure, both of which are directly related to RA-associated muscle loss (5). High disease activity promotes muscle catabolism through pro-inflammatory cytokines, while cumulative glucocorticoid use causes both muscle atrophy and metabolic alterations that subsequently affect albumin levels. The absence of these variables limits our ability to completely distinguish the effects of disease activity, treatment, and NPAR on muscle mass. Furthermore, the absence of detailed nutritional assessment data—including dietary protein intake, prealbumin, or transferrin levels—limits our ability to directly evaluate the nutritional contribution to NPAR’s association with LMM. Third, the definitions of LMM differed between the hospital cohort and NHANES: AWGS criteria were used in the hospital cohort, whereas EWGSOP-based criteria were used in NHANES, which may introduce heterogeneity. Fourth, RA status in NHANES was based on self-reported physician diagnosis, which may lead to potential misclassification. Fifth, the two cohorts show significant differences in racial composition, which may affect the interpretation of cross-cohort comparisons. Finally, although NPAR is clinically accessible, its optimal cutoff value requires further validation in prospective and multicenter RA cohorts.

## 5 Conclusion

This study demonstrates that elevated NPAR independently increases the risk of low muscle mass in RA patients, a core feature of sarcopenia, and is associated with increased all-cause mortality in this population. These findings highlight the clinical relevance of neutrophil-associated inflammatory burden in RA-related low muscle mass and suggest that NPAR may serve as a simple, inexpensive screening biomarker for early risk stratification and prognostic assessment. Future prospective studies are warranted to validate these findings, clarify causal mechanisms, and determine whether interventions targeting inflammation, nutrition, and muscle function can improve outcomes in RA patients with elevated NPAR.

## Data Availability

The raw data supporting the conclusions of this article will be made available by the authors, without undue reservation.

## Supplementary material

Supplementary Table 1 Univariate and multivariate logistic regression analysis.

Supplementary Table 2 Variance inflation factors (VIFs) for covariates in the multivariable logistic regression model.

Supplementary Table 3 Comparison of area under the ROC curve (AUC) for NPAR versus Its components and other inflammatory markers in predicting LMM.

Supplementary Table 4 Sensitivity analysis of the association between NPAR and sarcopenia risk in patients with RA after excluding those (n = 246) with clinical infection or markedly elevated CRP (>100 mg/L).

Supplementary Table 5 Firth penalized logistic regression analysis of the association between NPAR and LMM risk in the NHANES cohort.

Supplementary Table 6 Firth penalized Cox regression analysis of the association between NPAR and all-cause mortality **among** RA participants with LMM in the NHANES cohort (parsimonious model†).

Supplementary Figure 1 Kaplan-Meier survival curves according to NPAR levels among RA participants with low muscle mass in the NHANES cohort.
