# Supplementary material for: Neutrophil percentage-to-albumin ratio is associated with low muscle mass risk in rheumatoid arthritis: evidence from a hospital-based cohort and NHANES validation
Source: Front Immunol. 2026 Jul 31;17:1881390. doi: 10.3389/fimmu.2026.1881390 (PMC13473169; doi:10.3389/fimmu.2026.1881390)
Supplement: Supplementary file 1 [file Supplementaryfile1.pdf]

Supplementary Table S1. Univariate and multivariate logistic regression analysis.

Variables	Univariate Logistic Regression					Multivariate logistic regression				
	β	S.E	Z	P	OR (95% CI)	β	S.E	Z	P	OR (95% CI)
Gender										
Male					1.00 (Reference)					1.00 (Reference)
Female	-0.12	0.18	-0.64	0.522	0.89 (0.62 ~ 1.28)	-0.18	0.23	-0.76	0.447	0.84 (0.53 ~ 1.32)
Age										
≤39					1.00 (Reference)					1.00 (Reference)
40˜59	0.24	0.21	1.11	0.265	1.27 (0.84 ~ 1.92)	-0.15	0.42	-0.35	0.725	0.86 (0.38 ~ 1.96)
60˜79	-0.03	0.25	-0.11	0.916	0.97 (0.60 ~ 1.58)	-0.60	0.82	-0.74	0.462	0.55 (0.11 ~ 2.71)
≥80	-0.18	0.41	-0.43	0.664	0.84 (0.38 ~ 1.86)	-1.46	1.11	-1.32	0.187	0.23 (0.03 ~ 2.04)
BMI										
<18.5					1.00 (Reference)					1.00 (Reference)
18.5˜24.9	0.37	0.47	0.80	0.423	1.45 (0.58 ~ 3.63)	0.75	0.71	1.06	0.288	2.12 (0.53 ~ 8.48)
25˜29.9	0.44	0.48	0.92	0.359	1.55 (0.61 ~ 3.99)	0.88	0.94	0.93	0.353	2.40 (0.38 ~ 15.27)
≥30	0.62	0.67	0.93	0.352	1.86 (0.50 ~ 6.85)	1.10	1.36	0.81	0.417	3.01 (0.21 ~ 43.15)
Smoking										
No					1.00 (Reference)					1.00 (Reference)
Yes	-0.97	0.49	-2.00	0.046	0.38 (0.15 ~ 0.98)	-1.02	0.55	-1.84	0.066	0.36 (0.12 ~ 1.07)
Drinking										
No					1.00 (Reference)					1.00 (Reference)
Yes	-0.25	0.35	-0.72	0.470	0.78 (0.39 ~ 1.55)	0.12	0.60	0.21	0.836	1.13 (0.35 ~ 3.63)
Disease duration										
1-5					1.00 (Reference)					1.00 (Reference)
6-10	-0.09	0.30	-0.30	0.766	0.91 (0.50 ~ 1.66)	-0.05	0.43	-0.11	0.909	0.95 (0.41 ~ 2.19)

10˜20	0.96	0.28	3.44	<0.001	2.61 (1.51 ~ 4.52)	1.19	0.60	1.98	0.048	3.30 (1.01 ~ 10.76)
>20	1.78	0.37	4.81	<0.001	5.95 (2.88 ~ 12.30)	2.34	1.03	2.26	0.024	10.39 (1.37 ~ 78.75)
Diabetes										
No					1.00 (Reference)					1.00 (Reference)
Yes	-0.37	0.27	-1.35	0.178	0.69 (0.40 ~ 1.18)	-0.43	0.33	-1.32	0.188	0.65 (0.34 ~ 1.24)
Hypertension										
No					1.00 (Reference)					1.00 (Reference)
Yes	0.37	0.20	1.84	0.066	1.44 (0.98 ~ 2.13)	0.40	0.24	1.64	0.101	1.49 (0.92 ~ 2.41)
CVDs										
No					1.00 (Reference)					1.00 (Reference)
Yes	-0.19	0.24	-0.79	0.429	0.83 (0.52 ~ 1.32)	-0.14	0.29	-0.47	0.639	0.87 (0.49 ~ 1.54)
CKD										
No					1.00 (Reference)					1.00 (Reference)
Yes	-0.17	0.26	-0.64	0.519	0.85 (0.51 ~ 1.40)	-0.26	0.31	-0.84	0.400	0.77 (0.42 ~ 1.41)
ILD										
No					1.00 (Reference)					1.00 (Reference)
Yes	-0.62	0.45	-1.37	0.169	0.54 (0.22 ~ 1.30)	-0.63	0.58	-1.09	0.276	0.53 (0.17 ~ 1.65)
Dyslipidemia										
No					1.00 (Reference)					1.00 (Reference)
Yes	0.11	0.23	0.48	0.633	1.12 (0.71 ~ 1.76)	0.13	0.29	0.43	0.664	1.13 (0.64 ~ 2.00)
Osteoporosis										
No					1.00 (Reference)					1.00 (Reference)
Yes	1.45	0.19	7.67	<0.001	4.25 (2.94 ~ 6.15)	0.46	0.59	0.78	0.435	1.59 (0.50 ~ 5.08)
Stroke										
No					1.00 (Reference)					1.00 (Reference)

Yes	-0.27	0.28	-0.94	0.345	0.77 (0.44 ~ 1.33)	-0.37	0.34	-1.08	0.282	0.69 (0.35 ~ 1.35)
NSAIDs										
No					1.00 (Reference)					1.00 (Reference)
Yes	0.08	0.22	0.38	0.705	1.09 (0.70 ~ 1.68)	0.23	0.34	0.69	0.488	1.26 (0.65 ~ 2.45)
Glucocorticoids										
No					1.00 (Reference)					1.00 (Reference)
Yes	1.35	0.19	7.19	<0.001	3.85 (2.67 ~ 5.57)	-0.12	0.63	-0.18	0.854	0.89 (0.26 ~ 3.08)
Methotrexate										
No					1.00 (Reference)					1.00 (Reference)
Yes	0.02	0.18	0.11	0.911	1.02 (0.72 ~ 1.45)	0.22	0.22	1.02	0.310	1.24 (0.82 ~ 1.90)
Leflunomide										
No					1.00 (Reference)					1.00 (Reference)
Yes	0.03	0.24	0.15	0.883	1.04 (0.65 ~ 1.65)	0.07	0.29	0.23	0.820	1.07 (0.60 ~ 1.89)
Hydroxychloroquine										
No					1.00 (Reference)					1.00 (Reference)
Yes	0.35	0.29	1.22	0.221	1.42 (0.81 ~ 2.49)	0.35	0.35	0.99	0.322	1.42 (0.71 ~ 2.82)
Sulfasalazine										
No					1.00 (Reference)					1.00 (Reference)
Yes	-13.49	441.37	-0.03	0.976	0.00 (0.00 ~ Inf)	-13.72	469.89	-0.03	0.977	0.00 (0.00 ~ Inf)
Iguratimod										
No					1.00 (Reference)					1.00 (Reference)
Yes	-0.32	0.25	-1.27	0.206	0.73 (0.45 ~ 1.19)	-0.31	0.30	-1.05	0.293	0.73 (0.41 ~ 1.31)
Tofacitinib										
No					1.00 (Reference)					1.00 (Reference)
Yes	0.21	0.21	0.99	0.323	1.23 (0.81 ~ 1.87)	0.13	0.26	0.47	0.635	1.13 (0.67 ~ 1.91)

Baricitinib										
No					1.00 (Reference)					1.00 (Reference)
Yes	0.40	0.56	0.72	0.474	1.49 (0.50 ~ 4.42)	0.14	0.76	0.19	0.852	1.15 (0.26 ~ 5.06)
TNF-α inhibitor										
No					1.00 (Reference)					1.00 (Reference)
Yes	-0.07	0.26	-0.28	0.783	0.93 (0.56 ~ 1.56)	-0.09	0.32	-0.27	0.790	0.92 (0.49 ~ 1.73)
RF										
≤20					1.00 (Reference)					1.00 (Reference)
>20	0.58	0.25	2.32	0.021	1.78 (1.09 ~ 2.91)	0.75	0.49	1.53	0.126	2.11 (0.81 ~ 5.49)
ACPA(U/mL)										
0-5					1.00 (Reference)					1.00 (Reference)
5.1~200	0.53	0.32	1.63	0.103	1.69 (0.90 ~ 3.19)	0.78	0.52	1.50	0.133	2.18 (0.79 ~ 6.02)
>200	0.56	0.32	1.74	0.082	1.75 (0.93 ~ 3.30)	0.76	0.75	1.01	0.312	2.14 (0.49 ~ 9.34)
CRP(mg/L)										
0-8					1.00 (Reference)					1.00 (Reference)
＞8	0.05	0.22	0.21	0.831	1.05 (0.68 ~ 1.63)	-0.64	0.42	-1.52	0.129	0.53 (0.23 ~ 1.21)
ESR										
Normal					1.00 (Reference)					1.00 (Reference)
Higher	-0.24	0.23	-1.04	0.301	0.79 (0.50 ~ 1.24)	-0.15	0.29	-0.50	0.616	0.86 (0.49 ~ 1.53)
NPAR										
Low					1.00 (Reference)					1.00 (Reference)
High	1.16	0.19	6.00	<0.001	3.18 (2.18 ~ 4.64)	0.23	0.42	0.54	0.003	1.26 (1.01 ~ 2.87)
WBC	0.03	0.04	0.71	0.480	1.03 (0.95 ~ 1.13)	0.01	0.07	0.13	0.897	1.01 (0.88 ~ 1.16)
RBC	-0.03	0.13	-0.21	0.835	0.97 (0.75 ~ 1.26)	0.15	0.20	0.74	0.456	1.16 (0.79 ~ 1.71)
NEU	-0.06	0.02	-2.45	0.011	0.93(0.90 ~ 0.97)	-0.04	0.06	-0.65	0.442	0.85 (0.72 ~ 1.18)

NEU percentage	0.04	0.01	7.49	<0.001	1.04 (1.03 ~ 1.05)	0.06	0.06	1.05	0.295	1.06 (0.95 ~ 1.19)
PLT	-0.00	0.00	-0.47	0.640	1.00 (1.00 ~ 1.00)	-0.01	0.00	-2.24	0.056	0.99 (0.99 ~ 0.99)
MONO	0.28	0.59	0.48	0.630	1.33 (0.42 ~ 4.18)	0.44	0.89	0.50	0.618	1.56 (0.27 ~ 8.96)
LYM	0.18	0.12	1.45	0.148	1.20 (0.94 ~ 1.52)	0.83	0.27	3.03	0.052	2.29 (1.34 ~ 3.91)
DDi	0.00	0.00	1.25	0.210	1.00 (1.00 ~ 1.00)	0.00	0.00	1.31	0.189	1.00 (1.00 ~ 1.00)
TC	0.00	0.08	0.06	0.954	1.00 (0.86 ~ 1.18)	0.06	0.10	0.58	0.561	1.06 (0.87 ~ 1.30)
HDL	-0.26	0.23	-1.13	0.257	0.77 (0.49 ~ 1.21)	-0.43	0.30	-1.43	0.153	0.65 (0.36 ~ 1.18)
LDL	-0.17	0.11	-1.55	0.120	0.84 (0.68 ~ 1.05)	0.03	0.15	0.22	0.823	1.04 (0.76 ~ 1.40)
ALB	-0.05	0.02	-2.45	0.014	0.95 (0.91 ~ 0.99)	-0.06	0.07	-0.75	0.452	0.95 (0.82 ~ 1.09)
SII	0.01	0.00	4.56	<0.001	1.01 (1.01 ~ 1.01)	0.00	0.00	0.25	0.802	1.00 (1.00 ~ 1.00)
NLR	0.19	0.04	4.63	<0.001	1.21 (1.12 ~ 1.32)	-0.00	0.20	-0.00	0.997	1.00 (0.68 ~ 1.47)
AISI	0.01	0.00	4.55	<0.001	1.01 (1.01 ~ 1.01)	-0.00	0.00	-0.45	0.653	1.00 (0.99 ~ 1.00)
PLR	0.00	0.00	0.96	0.339	1.00 (1.00 ~ 1.00)	0.01	0.00	2.16	0.051	1.01 (1.01 ~ 1.02)
SIRI	0.36	0.09	4.06	<0.001	1.43 (1.20 ~ 1.70)	-0.01	0.47	-0.01	0.989	0.99 (0.39 ~ 2.50)

Supplementary Table S2. Variance inflation factors (VIFs) for covariates in the multivariable logistic regression model.

Variables	VIF
Age (years)	1.6
BMI (kg/m^2)	1.1
Smoking	1.1
Drinking	1.4
Gender	1.1
Disease duration	1.2
Diabetes	1.1
Hypertension	1.1
CVDs	1.1
CKD	1.1
ILD	1
Dyslipidemia	1.1
Stroke	1.1
RF (IU/mL)	1.4
CRP (mg/L)	1.3
ACPA (U/mL)	1.5
ESR (mm/h)	1.1
WBC ($x10^9/L$)	1.3
RBC ($x10^{12}/L$)	1.5
PLT($x10^9/L$)	1.2
NEU($x10^9/L$)	1.4
DDi (mg/L)	1.1
TC(mmol/L)	1.1
HDL(mmol/L)	1.2
LDL(mmol/L)	1.3
ALB(g/dL)	1.4
NSAIDs	1.5
Methotrexate	1
Leflunomide	1.1
Hydroxychloroquine	1.1
Sulfasalazine	1.1
Iguratimod	1.1
Tofacitinib	1.1
Baricitinib	1.1
TNF-α inhibitor	1.3
NSAIDs	1.1

Supplementary Table S3. Comparison of area under the ROC curve (AUC) for NPAR versus Its components and other inflammatory markers in predicting LMM.

Variables	AUC (95% CI)	P-value vs. NPAR*	Optimal Cutoff	Sensitivity (%)	Specificity (%)	PPV(%)	NPV(%)
NPAR	0.80 (0.76–0.84)	—	9.55	72.0	76.5	83.9	70.2
NEU%	0.69 (0.63-0.72)	<0.001	48.5	64.5	62.0	75.5	45.3
Albumin	0.57(0.52-0.62)	<0.001	37.85	39.3	48.0	68.3	21.7
CRP	0.52(0.48-0.57)	<0.001	78.48	57.4	52.0	77.3	30.0
ESR	0.52(0.47-0.57)	<0.001	25.5	56.0	48.5	69.0	35.8
NLR	0.63(0.58-0.68)	<0.001	3.237	73.8	50.3	80.9	40.2
SII	0.62(0.58-0.68)	<0.001	529.94	61.1	62	82.1	35.8

* indicates pairwise comparison using DeLong test, with NPAR as the reference.

Supplementary Table S4. Sensitivity analysis of the association between NPAR and sarcopenia risk in patients with RA after excluding those (n = 246) with clinical infection or markedly elevated CRP (>100 mg/L).

	**Model 1**		**Model 2**		**Model 3**	
	OR(95% CI)	P	OR(95% CI)	P	OR(95% CI)	P
NPAR(Total)	1.23(1.17~1.30)	<0.001	1.27(1.17~1.39)	<0.001	1.31(1.18~1.45)	<0.001
NPAR levels						
Low-NPAR	Ref.		Ref.		Ref.	
High-NPAR	5.56(3.26~9.47)	<0.001	7.01(3.22~15.27)	<0.001	8.76(3.31~23.20)	<0.001
P for Trend		<0.001		<0.001		<0.001

OR: odds ratio. CI: confidence interval.

Model 1: unadjusted.

Model 2: adjusted for age, sex, BMI, disease duration, smoking status, and alcohol consumption.

Model 3: further adjusted for comorbidities, medication use, and laboratory indicators, excluding VIF>5, albumin and neutrophil percentage to avoid bias.

Supplementary Table S5. Firth penalized logistic regression analysis of the association between NPAR and LMM risk in the NHANES cohort.

**Variable**	**Model 1** (unadjusted)	**Model 2** (adjusted for age, sex, race)	**Model 3** (fully adjusted†)
NPAR (continuous)	OR = 1.25 (1.10–1.41), P < 0.001	OR = 1.25 (1.09–1.43), P = 0.001	OR = 1.27 (1.08–1.48), P = 0.004
**NPAR quartiles**			
Q1 (lowest)	Reference	Reference	Reference
Q2	OR = 1.20 (0.35–4.10), P = 0.767	OR = 0.79 (0.21–2.85), P = 0.716	OR = 0.64 (0.17–2.35), P = 0.502
Q3	OR = 2.40 (0.68–8.43), P = 0.175	OR = 2.05 (0.56–7.45), P = 0.281	OR = 1.23 (0.27–5.60), P = 0.783
Q4	OR = 5.85 (2.02–16.93), P = 0.001	OR = 4.80 (1.73–13.30), P = 0.003	OR = 4.62 (1.56–13.68), P = 0.006
**P for trend**	< 0.001	< 0.001	< 0.001

† Model 3 adjusted for age, sex, race, BMI, PIR, education, marital status, smoking, drinking, physical activity, diabetes, hypertension, and hyperlipidemia – identical to original Model 3 but estimated with Firth penalization. All models include 375 participants and 57 sarcopenia events. OR = odds ratio; CI = confidence interval.

Supplementary Table S6. Firth penalized Cox regression analysis of the association between NPAR and all-cause mortality among RA participants with LMM in the NHANES cohort (parsimonious model†).

Variable	**Model 1** (unadjusted)	**Model 2** (adjusted for age, sex, race, BMI)
NPAR (continuous)	HR = 1.10 (1.02–1.18), P = 0.017	HR = 1.11 (1.02–1.21), P = 0.018
**NPAR quartiles**		
Q1 (lowest)	Reference	Reference
Q2	HR = 2.00 (0.84–4.78), P = 0.119	HR = 1.85 (0.80–4.28), P = 0.152
Q3	HR = 1.73 (0.74–4.05), P = 0.209	HR = 1.48 (0.56–3.92), P = 0.427
Q4	HR = 2.68 (1.25–5.74), P = 0.011	HR = 2.31 (1.08–4.94), P = 0.031
**P for trend**	< 0.001	< 0.001

† Due to the small number of deaths (n = 21), we used a parsimonious model adjusting only for age, sex, race, and BMI, together with Firth penalization to reduce bias. Model 1 is unadjusted. HR = hazard ratio; CI = confidence interval.

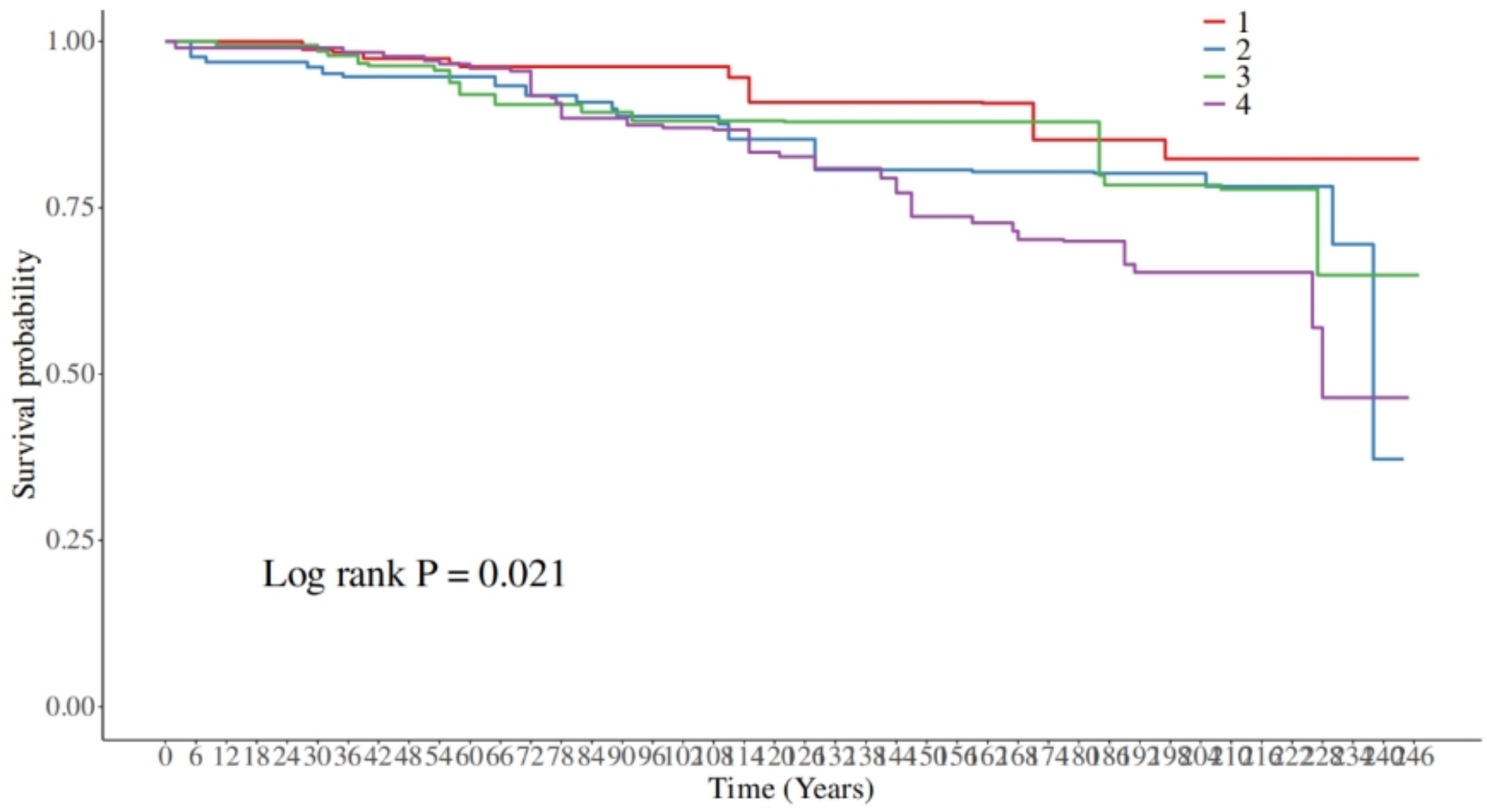

Supplementary Figure S1. Kaplan-Meier survival curves according to NPAR levels among RA participants with low muscle mass in the NHANES cohort.
